# The unique structural and functional characteristics of glomerular endothelial cell fenestrations and their potential as a therapeutic target in kidney disease

**DOI:** 10.1152/ajprenal.00036.2023

**Published:** 2023-07-20

**Authors:** Natalie C. Finch, Chris R. Neal, Gavin I. Welsh, Rebecca R. Foster, Simon C. Satchell

**Affiliations:** ^1^Bristol Renal, University of Bristol, United Kingdom; ^2^Langford Vets, University of Bristol, United Kingdom

**Keywords:** endothelial, fenestrations, filtration, function, glomerular

## Abstract

Glomerular endothelial cell (GEnC) fenestrations are a critical component of the glomerular filtration barrier. Their unique nondiaphragmed structure is key to their function in glomerular hydraulic permeability, and their aberration in disease can contribute to loss of glomerular filtration function. This review provides a comprehensive update of current understanding of the regulation and biogenesis of fenestrae. We consider diseases in which GEnC fenestration loss is recognized or may play a role and discuss methods with potential to facilitate the study of these critical structures. Literature is drawn from GEnCs as well as other fenestrated cell types such as liver sinusoidal endothelial cells that most closely parallel GEnCs.

## INTRODUCTION

**Figure 1.**
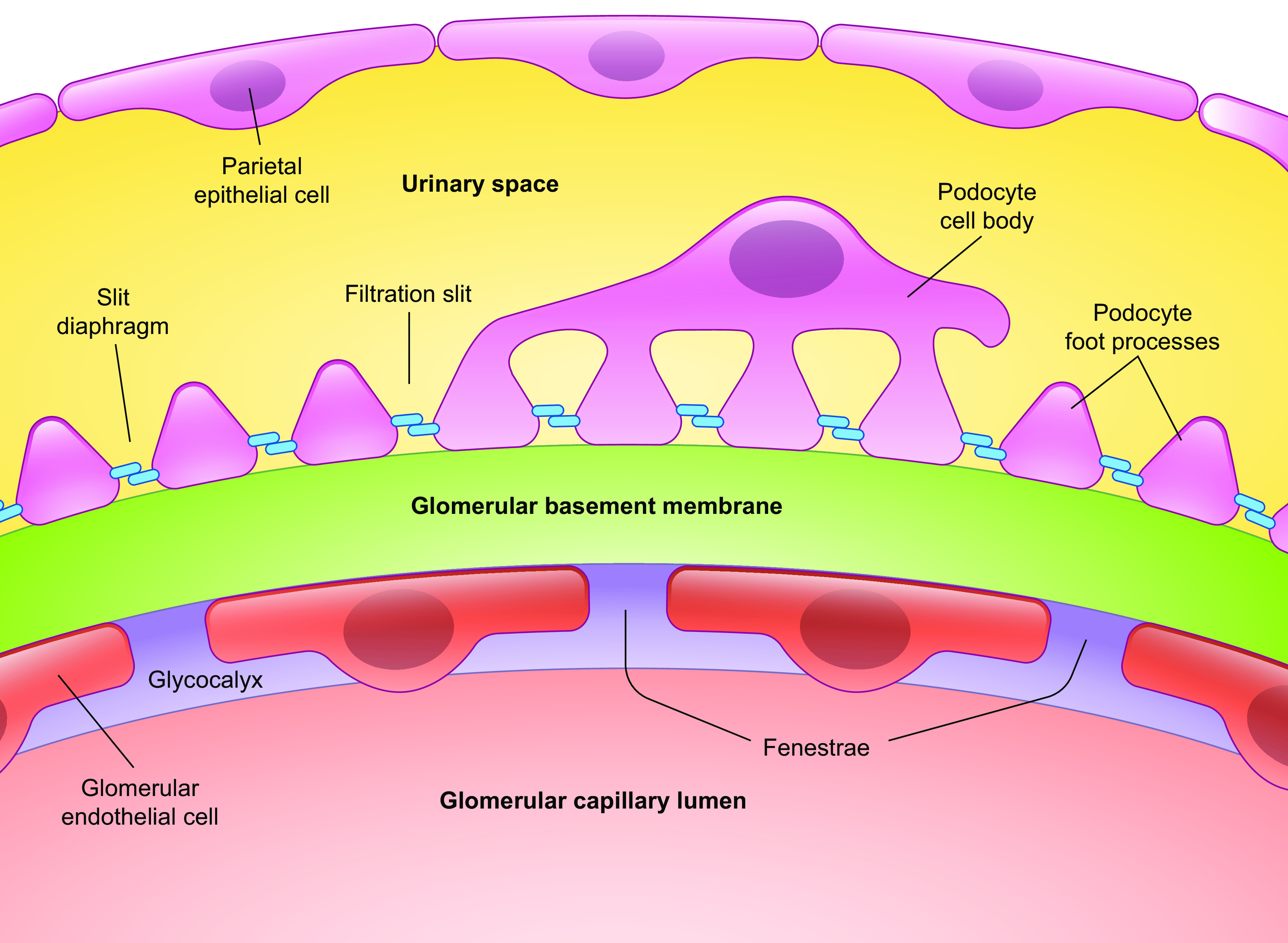
Diagram of the glomerular filtration barrier. GBM, glomerular basement membrane.

**Figure 2.**
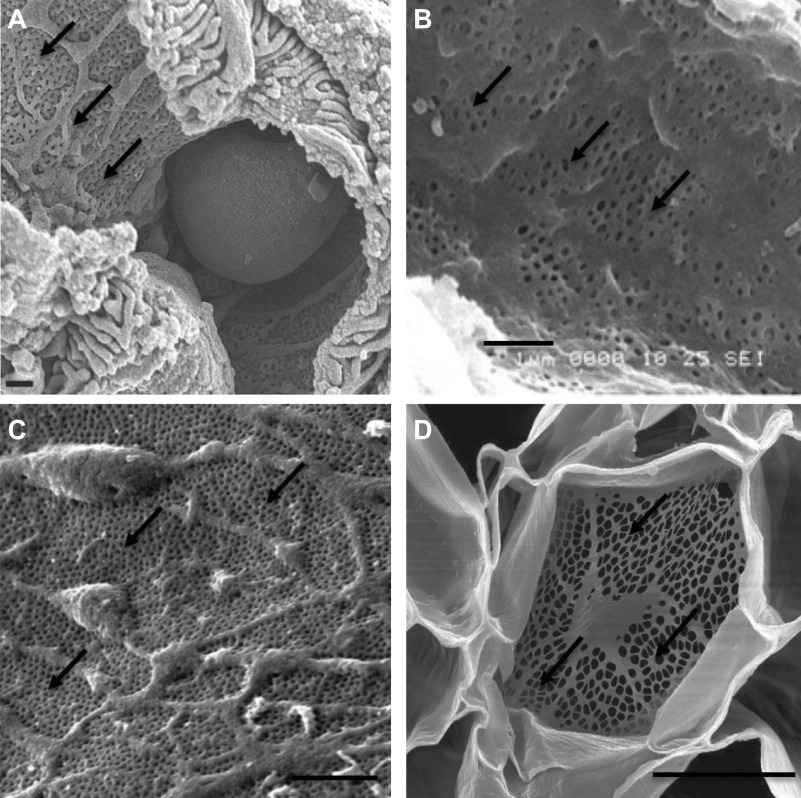
Scanning electron micrographs illustrating the fenestrated endothelium in the glomerular capillary surrounded by podocyte foot processes (*A*) (1), liver sinusoidal endothelial cells (*B*) (2), peritubular capillary (*C*) (3), and phloem system of *Ricinus communis* (castor bean; *D*) (4). Arrows indicate fenestrations in sieve plates. Scale bars = 1 µm in *A*–*C* and 20 µm in *D*.

The renal endothelium is highly heterogeneous, consisting of fenestrated and continuous endothelial cells. Glomerular endothelial cells (GEnCs) are highly differentiated cells lining the glomerular capillaries and forming the first layer of the glomerular filtration barrier (Fig. 1). GEnCs are perforated with nondiaphragmed fenestrations, in contrast to the peritubular capillary and ascending vasa recta endothelial fenestrae, which are diaphragmed (Fig. 2). The conduit vessels of the renal microcirculation such as the arterioles and venules and the descending vasa recta are composed of continuous endothelium (3). The focus of this review article is the GEnC fenestrations. Here, the characteristics of GEnC fenestrations and how this relates to their biology in health and disease are described. Current knowledge and knowledge gaps regarding their regulation are discussed.

The biology and function of GEnCs are highly specialized, with genetic analysis revealing their unique features relative to other endothelial cell types (5). GEnCs are perforated with transcytoplasmic pores known as fenestrations. Fenestrated endothelia exist in microcirculatory beds, where they are required to enable a high rate of fluid exchange between intra- and extravascular compartments such as the kidneys, small intestines, and endocrine glands. GEnC fenestrations allow the unimpeded passage of water and small dissolved solutes from the glomerular capillary lumen through endothelial cells, without the need for endocytosis or receptor-mediated mechanisms. Thus, they provide the first step in urine formation. GEnC fenestrations appear to be well conserved across species, including humans (6), birds (7), amphibians (8), and fish (9), although they may vary in size and distribution. Fenestrations are even present in the phloem vascular system of plants (Fig. 2) (4). Within endothelial cells, they are located in the thin cytoplasmic extensions of the cell and exist mainly in organized clusters known as sieve plates reflecting their biological role (6). In GEnCs, the fenestrae cover ∼50% of the glomerular capillary surface area (10). They are hourglass in shape, with the smallest diameter of the fenestration being midway between the apical and basal surfaces (11). Therefore, at the base of the fenestration, the diameter is much larger and, hence, the actual area of the basement membrane available for filtration at the base of the fenestrae is greater than that of the smallest diameter (83% vs. 20% in rats; 11). This is an important consideration when visualizing fenestrations and reporting width or coverage measurements.

## KEY ULTRASTRUCTURAL CHARACTERISTICS OF GEnC FENESTRAE THAT DEFINE THEIR FUNCTION

### The Absence of Diaphragms

The majority of fenestrated endothelia possess diaphragms that span the pore. GEnC fenestrations are unique in that quiescent cells generally do not possess such a diaphragm, with diaphragmed fenestrae being present in only 2% of total glomerular capillary cross sections in rats (12). Differences between afferent and efferent arterioles of the glomerulus have been noted, with afferent arterioles possessing nondiaphragmed fenestrae and efferent arterioles possessing diaphragmed fenestrae (13). Of the fenestrated endothelia, GEnCs most closely parallel liver sinusoidal endothelial cells (LSECs; Fig. 2), which also possess nondiaphragmed fenestrations (6), with an important distinction being that LSECs are discontinuous and hence lack a basement membrane. The lack of diaphragms has functional significance. In LSEC fenestrations, it allows bidirectional transfer of small or soluble substrates between blood and the extracellular space of Disse (14), with LSEC fenestrae being critical conduits of lipoproteins (15) and pharmacological agents (16). In GEnC fenestrations, the lack of diaphragms enables the high fluid flux driven by hydrostatic pressure through the fenestrations with the resistance to water permeability being negligible (17). The presence of a diaphragm in other fenestrated endothelial cell types is speculated to provide more controlled permeability (18).

A universal component of fenestral diaphragms is plasmalemmal vesicle-associated protein-1 (PLVAP), and its presence positively identifies a diaphragm within a fenestration. Reports of glomerular PLVAP expression have varied from complete absence up to 5% (12, 18, 19). In mature quiescent GEnCs, PLVAP expression is consistently low. The number of diaphragmed fenestrae is approximately four times greater in GEnCs recovering from injury (12). The presence of increased numbers of diaphragmed fenestrations has also been reported in diabetic mice, suggesting pathological injury (20). Diaphragmed fenestrae are present in the later embryonic stages of GEnC (12, 19) and LSEC (21) development but are lost within 2 wk of birth in rat kidneys (22). These findings suggest that the presence of a diaphragm may play a role in remodeling during development and following injury. Crucially, PLVAP knockout mice had normal fenestrae formation compared with control mice, indicating that PLVAP is not essential for the formation of mature fenestrae (18). Furthermore, PLVAP loss does not impair kidney function or result in structural changes in the kidney (18).

### The Endothelial Glycocalyx

The endothelial glycocalyx lines the luminal side of the GEnC and is also present within the fenestrations (23). The intrafenestral glycocalyx composition differs from that on the GEnC surface, having a higher concentration of heparan sulfate (24). This may be important for the permeability properties of fenestrae. GEnC fenestrations are reported to be ∼70 nm in diameter; however, owing to the presence of the glycocalyx, their effective pore size is considered to be lower (25). Mathematical modeling also suggests that the glycocalyx contributes to the permeability properties of the pore (26). The GEnC fenestral pore size is much greater than the size of an albumin molecule, which is ∼3.8 × 15 nm (27). This led to the assumption that GEnC fenestrae did not provide significant hindrance to albumin passage. However, the presence of the glycocalyx within the fenestration is now known to be important in restricting albumin (6). Whether the glycocalyx provides any regulatory or structural support role to GEnC fenestrae remains to be studied.

## REGULATION OF FENESTRATIONS

LSEC fenestrae are considered to be dynamic structures, responding to circulating stimuli and local factors that regulate their diameter and number (28). Relative to other endothelial cell types such as LSECs, understanding of the fundamental biology of GEnC fenestral regulation is deficient. Little is known about the regulatory and structural proteins involved in their maintenance.

### The Immature Glomerulus

Current thoughts suggest that the glomerular endothelium develops from circulating hemangioblasts that are recruited into the kidney via angiogenic factors and become closely associated with mesangial cells and podocytes (3). This process is driven by vascular endothelial growth factor (VEGF)-A acting as a chemoattractant. In the early embryonic immature glomerulus, GEnCs lack fenestrae (12). As the embryonic kidney matures, fenestrae that are predominantly diaphragmed develop (12). This is promoted by VEGF-A (29). GEnCs in mice lacking endothelial a disintegrin and metalloprotease domain 10 (ADAM10), a key regulator in Notch signaling, demonstrated persistence of an immature glomerular endothelium including the presence of diaphragmed fenestrae (30). In this mouse model, 65.7% of glomeruli possessed diaphragmed fenestrae compared with 6.4% of control mice and there was upregulation of PLVAP expression, the main component of fenestral diaphragms (30). Importantly, morphology of other glomerular structures such as podocytes and the glomerular basement membrane (GBM) were comparable between ADAM10-deficient and control mice. These findings suggest an essential role of ADAM10 and Notch signaling in maturation of GEnC fenestrae with an arrested immature state in mice lacking ADAM10. No functional consequences of this were noted, although specific measurement of the permeability of the filtration barrier was not performed.

### VEGFs in GEnC Fenestration Regulation

VEGF-A has a critical role in GEnC fenestration regulation, and mice with podocyte-specific VEGF-A knockout demonstrate decreased fenestrations (31, 32). In the healthy glomerulus, VEGF-A is produced by podocytes and interacts with VEGF receptor 2 (VEGFR2) on GEnCs to induce downstream signaling pathways. Studies of VEGF-A/VEGFR2 pathway manipulations have highlighted that this angiogenic factor, and specifically podocyte-derived VEGF-A, is required for a healthy glomerular endothelial phenotype (33). VEGF-A is known to induce fenestrations in many other endothelial cells, with a particular role in fenestration formation in tumor endothelial cells (34). In a model of membranoproliferative glomerulonephritis, podocyte injury results in reduced VEGF-A production leading to GEnC fenestration loss (35). VEGF-A exists as different isoforms denoted by the number of amino acids, with VEGF_121_ being the predominant isoform in human glomeruli and VEGF_164_ in mouse glomeruli (36). Angiogenic (a) and antiangiogenic (b) splice variants of VEGF_165_ are also recognized (37). Glomerular overexpression of the antiangiogenic isoform, VEGF_165_b, decreases fenestration density and width (38). An intricate balance of both angiogenic and antiangiogenic VEGF_165_ is likely to be required to maintain GEnC fenestrae. In podocyte-specific overexpression of VEGF-A, the number of diaphragmed fenestrae (determined by PLVAP expression) is decreased. Conversely, in podocyte-specific VEGF-A knockdown, there is increased PLVAP expression (13). This suggests that VEGF-A not only maintains fenestrations but also plays a role either in the development of diaphragmed fenestrae or in contributing to GEnC injury resulting in the development of diaphragmed fenestrae. Overexpression of VEGF-A in tubular epithelial cells increases serum VEGF-A concentrations, a mechanism proposed to expose GEnCs to higher VEGF-A concentrations via the systemic circulation (13). Tubular ablation is suggested to contribute to reduced circulating VEGF-A, resulted in increased glomeruli PLVAP, suggesting the development of diaphragmed fenestrae. A model for VEGF-dependent plasticity and the effect of endothelial cell morphology has been proposed (13). This model suggests that very high VEGF-A concentrations are required for the formation of nondiaphragmed fenestrae with intermediate VEGF-A concentrations associated with diaphragmed fenestrae (13).

Current evidence suggests that VEGF-A regulates fenestrations through promoting actin cytoskeleton remodeling and cell membrane lipid rafts (39). VEGF-A is known to be important in both neovascularization and vascular permeability; however, the downstream signaling pathways for these appear to be different. The GTP-binding protein Rac is key in VEGF-A downstream signaling pathways to induce fenestrations and hence increase permeability (40), although this remains to be studied in the glomerulus. VEGF-A also activates ERK1/2 pathways, and this is important in maintaining a healthy endothelium, with ERK1/2 knockout mice demonstrating loss of GEnC fenestrations (41).

VEGF-C is a lymphangiogenic growth factor also produced by podocytes and signals via VEGF receptor 3 (VEGFR3). It has been suggested that the expression of VEGF-C may differ in healthy and injured kidneys (42). VEGFR3 expression in the glomerular endothelium is highest during early kidney development but remains detectable through maturation (42). It has recently been reported that the functional role of VEGFR3 in glomerular capillary development is independent of VEGF-C, unlike in the lymphatic endothelium (42). However, although VEGF-C overexpression did not alter GEnC fenestration density in normal mice, it prevented fenestration loss in type 1 diabetic mice (43). Endothelium-specific deletion of VEGFR3 during development results in lack of GEnC fenestrations (42). VEGFR3 may play a unique role in regulating GEnC potentially via modulation of VEGFR2 signaling, as VEGFR3 knockout did not result in defects in kidney peritubular capillaries (42).

### Caveolin-1 and Aldosterone

Caveolin-1 is the predominant structural protein in caveolae and has been associated with fenestral porosity and diameter in LSECs (44). However, both GEnC (45) and LSEC (46) fenestrations appear normal in caveolin knockout mice (45). Caveolin is expressed in GEnCs (47) but not in fenestrae (45). Caveolin interacts with mineralocorticoid receptors (MRs), and binding of aldosterone to the caveolin-MR complex induces oxidative signaling cascades (48). In LSECs, increased aldosterone resulted in fenestration loss through caveolin-mediated oxidation pathways (48). Furthermore, fenestration loss was rescued with the aldosterone antagonist spironolactone (48). A subsequent study demonstrated that autophagy in LSECs resulted in caveolin-1 degradation and promotion of fenestration loss (49). Treatment with the autophagy inhibitors 3-methyladenine or VEGF-A prevented autophagic caveolin-1 degradation and maintained fenestrations (49). The evidence suggests that caveolin-1 is not required for GEnC fenestration formation, but a role in regulating fenestrations in mature GEnC is plausible considering the mechanistic relationship between caveolin-1 and aldosterone in LSECs. Aldosterone has been shown to induce glomerular injury (50), but fenestration loss has not been reported in mice treated with aldosterone (51). The effect of chronic aldosterone increases on GEnC has not been reported.

### Eps15 Homology Domain-Containing Protein 3

**Figure 3.**
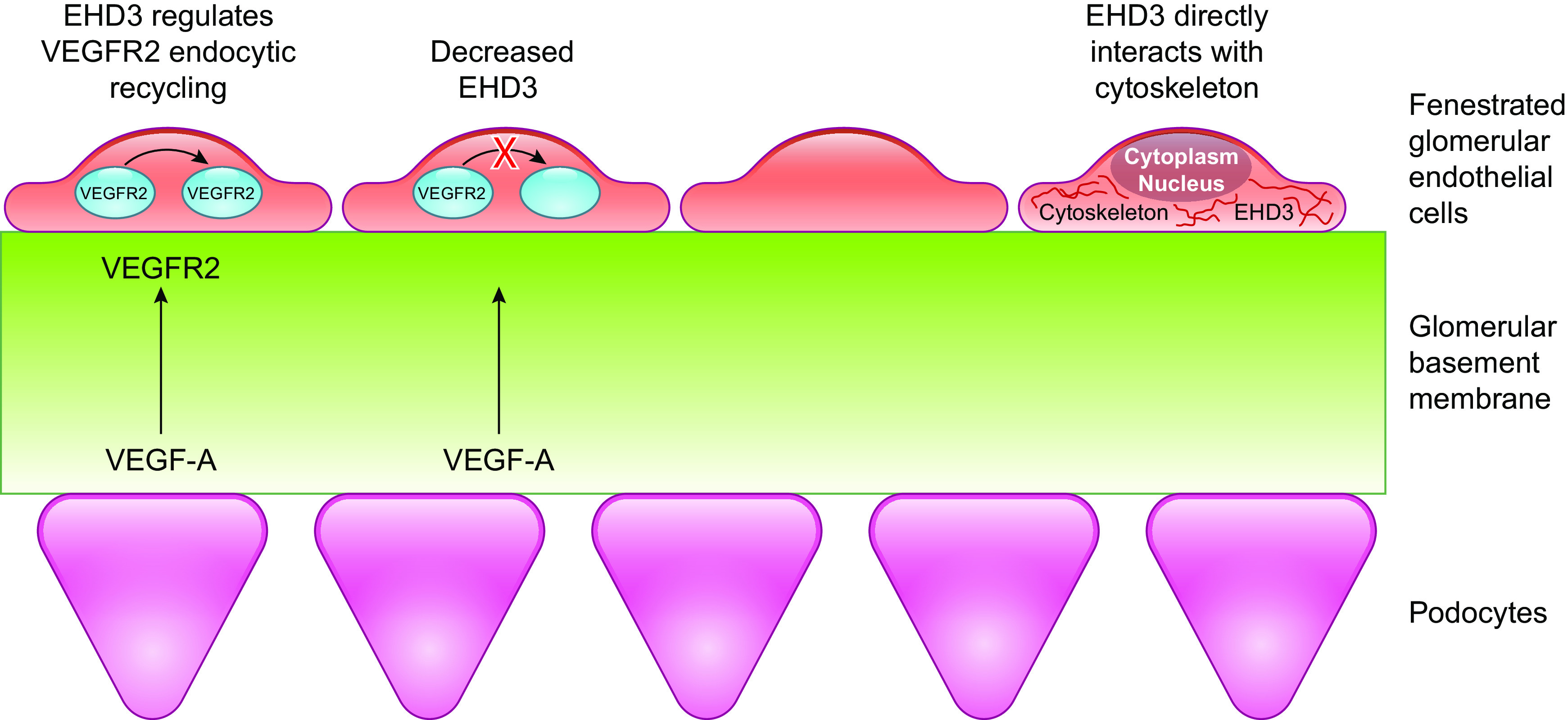
Postulated mechanism of fenestration regulation by Eps15 homology domain-containing protein (EHD)3 via altered vascular endothelial growth factor (VEGF) signaling. VEGFR2, VEGF receptor 2.

Eps15 homology domain-containing protein (EHD)3 is an endosomal transport protein (52). Within the kidney, EHD3 is specifically expressed in GEnCs (53–55) and has been localized to the fenestrae (53). Single-cell transcriptome profiling also demonstrated that within the glomerulus, EHD3 expression is high in fenestrated GEnCs compared with nonfenestrated arteriolar (afferent and efferent) cells (56). Knockout of EHD3 (with EHD4) results in the development of kidneys with absence of GEnC fenestrations (57). The transcription factor *Tbx3* is important in the development of glomerular capillaries, with knockout of this factor resulting in downregulation of EHD3 expression and fenestration loss (55). Decreased glomerular EHD3 expression has also been reported in diabetes, chronic kidney disease, and focal segmental glomerulosclerosis (58). In vitro knockdown of EHD3 decreases fenestration formation in a fenestration-forming endothelial cell line, mouse brain endothelioma “bEND5” cells (58). Due to the role of EHD3 in endocytic trafficking, it has been postulated that it may regulate recycling and membrane availability of VEGFR2, resulting in altered VEGF signaling (Fig. 3; 57, 58). It is also possible that EHD3 regulates GEnC fenestrations via other mechanisms, such as indirectly via endocytic recycling of other angiogenic factors or directly via interactions with the cellular cytoskeleton. However, EHD3 does not associate with actin microfilaments, and treatment with cytochalasin D, an actin polymerizing inhibitor, does not result in altered EHD3 cellular location (59).

## GEnC FENESTRAE BIOGENESIS

### Role of the Actin Cytoskeleton

**Figure 4.**
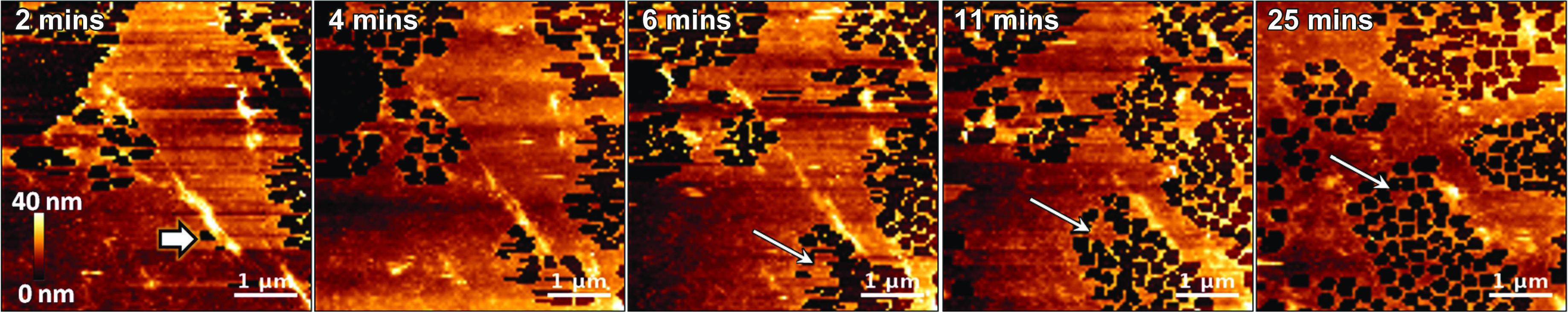
Topographical images using atomic force microscopy of live liver sinusoidal endothelial cells treated with 21 µmol/dm^3^ of cytochalasin B and obtained at 2, 4, 6, 11, and 25 min following treatment. An increase in fenestration density (white arrows) was observed following cytochalasin B treatment, with newly formed sieve plates appearing within 2 min (64).

The formation of fenestrae in LSECs results from fusion of opposing plasma membranes (60) associated with actin cytoskeleton reorganization (61, 62). Structured illumination microscopy (SIM) imaging of the underlying actin cytoskeleton in LSECs reveals bands of F-actin surrounding the sieve plates (63). Administration of an actin cytoskeleton-reorganizing agent, cytochalasin B, results in rapid widening of existing sieve plates and an increased number of fenestrations over 25 min, with newly formed sieve plates observed after 2 min (Fig. 4; 64). Fenestration formation is also induced following actin depolymerization and reorganization of the actin cytoskeleton using latrunculin A in a brain endothelioma cell line (65). A fusion pore formed by the merger of plasma membrane with vesicles in the process of exocytosis is opened in 20 min in pituitary cells (66). The mechanism of fenestration formation is suggested to be similar in GEnCs (67), with actin cytoskeleton rearrangement followed by fusion of the basolateral and apical plasma membrane. However, because of the relative attenuation of the cell cytoplasm in fenestrated areas, a fenestration rather than a fusion pore is formed. Extrapolating from other cell types, a timeframe of minutes provides a likely estimate for the rate of fenestration formation.

Actin cytoskeleton reorganization is presumed to play a key role in fenestration regulation. At the cytoskeletal level, the regulation of fenestration formation has been studied in detail in bEND5 cells (68), with fodrin also reported to be a key component of the cytoskeleton required for fenestration formation. Ezrin, radixin, and moesin (ERM) proteins interact with transmembrane proteins and the underlying actin cytoskeleton (69). Moesin is the predominant ERM protein in GEnCs (70). These ERM proteins are proposed to regulate cell architecture including fenestrae via actin remodeling. Moesin was present in fenestration-rich areas of the cell membrane in bEND5 cells and positively regulated fenestration formation (68). Annexin II, a further membrane-associated actin-binding protein, was found to negatively regulate fenestration formation in bEND5 cells (68). The Cl− intracellular channels CLIC5A and CLIC4 are expressed in GEnCs in vivo (71), with CLIC5A localizing to the fenestrated area of GEnCs (72). CLIC5A and CLIC4 activate ERM phosphorylation with reduced phosphorylation in mice deficient in CLIC5A or CLIC4 (71). Furthermore, in older mice (∼8 mo) with dual knockout of CLIC5A and CLIC4, there is not only decreased GEnC ERM protein phosphorylation but also marked loss of GEnC fenestrae (71). Thus, it appears likely that ERM proteins play a key role in cytoskeleton reorganization and the regulation of GEnC fenestrae.

### Sieve Raft Hypothesis

**Figure 5.**
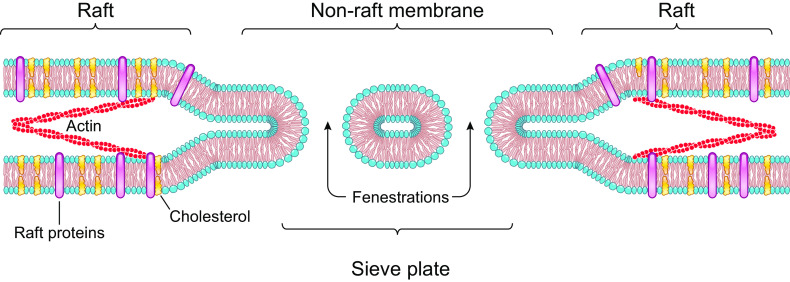
Schematic representing the sieve raft hypothesis for the formation of fenestrations in liver sinusoidal endothelial cells. This hypothesis proposes that fenestrations form within the nonraft microdomain but do not form in the raft domain in which the lipid rafts and actin provide membrane stability. (Adapted from Ref. 60.)

A recently proposed novel mechanism involved in the formation of fenestrations in LSECs involves the lipid raft structure of the cell membrane and has been termed the sieve raft hypothesis (Fig. 5) (60). Lipid rafts are specialized membrane microdomains composed of sphingolipid, cholesterol, and proteins. They provide membrane stability and organizing centers for membrane proteins and receptors. The actin cytoskeleton and lipid rafts are connected and stabilized through protein complexes such as ERM proteins and stabilin (60). Studies in LSECs have shown an inverse distribution between fenestrations and membrane lipid rafts, with fenestrations forming in nonlipid raft microdomains (60). The process of cell membrane bending and fusion in fenestration formation requires extensive deformations of the lipid bilayers and can only occur when lipid rafts are depleted (60). In the lipid raft microdomain, the actin cytoskeleton and lipid raft connection acts to maintain membrane stability and prevents fenestration formation. Cytochalasin D reduces lipid rafts in LSECs and increases fenestration formation (14). It is further suggested that agents that act upstream of the actin cytoskeleton, such as cytochalasin D, will influence fenestration density, whereas agents that can act directly on the lipid raft, such as 7-ketocholesterol, can additionally influence fenestration diameter (39). The sieve raft hypothesis and relationship between lipid rafts and fenestrations remain to be examined in GEnCs.

## HOW DO GEnCs REGENERATE AND FORM FENESTRATIONS FOLLOWING INJURY?

In the healthy kidney, GEnC turnover is low, with cell tracking studies demonstrating no change in cell number or distribution over 10 days (73). In response to VEGF or injury, a 4.4-fold increase in GEnC number was reported, with rates highest in the first 10 days but with clonal clusters still present 1–2 mo posttreatment (73). Bone marrow, circulating cells, and local tissue have been postulated to be the source of GEnC progenitor cells (74–76). In the liver, bone marrow-derived sinusoidal endothelial progenitor cells repair lost or injured LSECs (77). However, more recent evidence suggests that extrarenal progenitor cells are not involved in GEnC repair, indicating that local mechanisms within the kidney are responsible (73, 78). Angiogenesis with endothelial cells arising from preexisting blood vessels may play a role (79). Nonetheless, the proliferation of surviving GEnC following renal injury only appears to partially account for GEnC regeneration (78), suggesting that intrarenal progenitor niches must be involved (80). In response to VEGF or injury, cell tracking studies have demonstrated clonal expansion of local endothelial progenitor cells at the single-cell level in the same blood vessel (73). This proliferation appears to develop at the glomerular vascular pole (juxtaglomerular, afferent, and efferent arteriole segments; 73). The role of renin-producing juxtaglomerular cells as endothelial progenitor cells has been studied (80). Following endothelial cell injury, renal renin lineage cells invade the glomerulus during the repair phase to become intraglomerular cells and lose their ability to produce renin. However, these repopulating renal renin lineage cells do not contribute to endothelial cell replacement but rather develop into intraglomerular mesangial cells (80). Furthermore, mature GEnCs do not appear to be of the renal renin cell lineage (81). Transfer of autologous circulating endothelial progenitor cells has also been demonstrated to restore renal function in chronic experimental renovascular disease (82). No studies exploring the source of endothelial progenitor cells have reported the ultrastructural characteristics of GEnCs, including the presence of diaphragmed or nondiaphragmed fenestrae. What is evident is that, similar to the immature glomerulus, GEnCs in the postinjury state also demonstrate a greater percentage of diaphragmed fenestrae than controls (12) postulated to reflect a process of restorative reconstruction.

Renal renin-producing lineage cells may not be the source of glomerular endothelial progenitor cells; however, they have been suggested to play a role in fenestration regulation through the production of angiogenic factors including VEGF-A (83). An initial study demonstrated that downregulation of renin production in juxtaglomerular cells via G_s_α (a subunit of a membrane receptor catalyzing intracellular production of cAMP) resulted in endothelial injury (83). A further study subsequently showed that it is not reduced renin production per se that causes GEnC damage but rather the change in the renin-producing cell phenotype to a profibrotic phenotype as a result of G_s_α knockdown (84). This change in cell phenotype could contribute to GEnC fenestration dysregulation via alterations in the production of angiogenic factors. Indeed, increased glomerular PLVAP staining was present in G_s_α knockdown mice, suggesting increased numbers of diaphragmed fenestrations associated with immature or injured GEnCs (84).

## GEnC FENESTRATIONS IN DISEASE

As GEnC fenestrae are major contributors to hydraulic permeability at the filtration barrier, they will influence glomerular filtration rate (GFR). Their loss is associated with disease pathogenesis and decline in renal filtration function in multiple kidney diseases. Furthermore, decreased GEnC fenestration density is independently associated with declining GFR in humans (85). Notably, there is emerging evidence that endothelial cell injury and potentially endothelial cell fenestration loss may be one of the earliest changes in glomerular disease (86). Fenestration loss and GEnC injury may initiate podocyte damage via cross talk mechanisms, challenging the traditional concept that podocyte injury is the primary event in nephropathies (86).

### Diabetic Nephropathy

Glomerular endothelial injury and dysfunction are recognized in diabetic nephropathy (87). Abnormal angiogenesis including abnormal intraglomerular capillaries and new vessel formation (88) may contribute to glomerular hypertrophy and hyperfiltration in the early stages of diabetic nephropathy. By conjecture, it is possible that the GEnCs that comprise these vessels may have higher fenestration density or more readily form fenestrae, thus further contributing to hyperfiltration. The ultrastructural changes in the glomerulus associated with the later stages of diabetic nephropathy in which filtration function is beginning to decline include loss of fenestrations (89). Furthermore, in diabetes, decreased filtration function is not only associated with loss of fenestration density but also the development of diaphragmed fenestrae, suggesting fenestration loss and diaphragm formation as mechanisms of GFR loss in diabetic nephropathy and therefore as potential therapeutic targets (58). Glomerular VEGF-A expression is increased in early-stage diabetic nephropathy (90) but decreases in late-stage disease (91). Given that VEGF-A plays a critical role in regulating fenestrations, findings of GEnC fenestration changes in diabetes are unsurprising.

Factors proposed to play a role in the development of diabetic nephropathy include glucose-induced oxidative stress and glucose toxicity, both of which are associated with glomerular endothelial dysfunction (92, 93) that could result in fenestration loss. Serum concentrations of tumor necrosis factor (TNF) receptors 1 and 2 are inversely correlated with the percentage of fenestrated GEnCs in patients with type 2 diabetes, and this correlation is stronger than with other glomerular morphometric measurements such as glomerular basement membrane thickness (94). This suggests that a proinflammatory state in diabetes may contribute to GEnC fenestration loss. Renin-angiotensin system activation is also considered to play a role in the pathogenesis of diabetic nephropathy. Angiotensin expression is increased in cultured GEnCs exposed to high glucose conditions (95). Furthermore, increased GEnC fenestral width in diabetic rats is prevented by treatment with an angiotensin receptor blocker (95). Treatment with a Na^+^-glucose cotransporter 2 inhibitor in diabetic mice prevented GEnC fenestration loss and reduced PLVAP expression (considered a marker for diaphragm formation) via regulation of podocyte-derived VEGF-A (96). Activity of the enzyme xanthine oxidoreductase is also increased in diabetic kidney disease (97). Administration of topiroxostat, a nonpurine-selective xanthine oxidase (XO) inhibitor, prevented fenestration loss in diabetes, suggesting potential as a protective therapeutic (97).

GEnC fenestration loss has been shown to promote the development of diabetic nephropathy in patients with type 2 diabetes (89). It is further suggested that cross talk between damaged GEnCs and podocytes contributes to the development and progression of diabetic nephropathy (98, 99). Mitochondrial oxidative stress-induced GEnC injury is emerging as a key pathological mechanism in diabetic nephropathy. Endothelin-1 receptor type A-induced endothelial mitochondrial oxidative stress was demonstrated to result in GEnC injury and fenestration loss in diabetes (100). Furthermore, treatment with a mitochondria-targeted potent antioxidant prevented GEnC fenestration loss and also the characteristic morphological and functional changes associated with diabetic nephropathy in diabetic mice (100). Mitochondrial oxidative stress induces GEnC dysfunction that promotes podocyte injury (101). Evidence for GEnC cell injury preceding podocyte damage has also been demonstrated in an adriamycin-induced nephropathy model (102), further supporting the concept of GEnC injury and fenestration loss as early changes contributing to podocyte damage via cross talk mechanisms.

Aberrant cross talk between GEnCs and mesangial cells is also considered to play a role in endothelial-to-mesenchymal transition in endothelial injury, contributing to the development of sclerotic glomerular diseases such as diabetic nephropathy. Integrin-α_8_ (ITGA8), expressed exclusively on mesangial cells, is important in this process through its regulation of transforming growth factor (TGF)-β (a known mediator of endothelial-to-mesenchymal transition) bioavailability (103).

Insulin undergoes renal clearance, being freely filtered at the glomerular filtration barrier (104). Insulin binding to the insulin receptor in hepatocytes similarly requires its passage from the blood across the liver sinusoidal endothelium. Loss of LSEC fenestrations reduces the ability for insulin to pass through the endothelium and contributes to hyperinsulinemia, insulin resistance, and impaired hepatic insulin signaling (105). Furthermore, insulin uptake by the liver is reduced by 30% in old mice with decreased LSEC fenestration density and 20% in mice treated with a defenestrating agent (105). It is therefore plausible that GEnC fenestration loss may result in decreased filtration of insulin contributing to hyperinsulinemia and decreased insulin receptor signaling in the kidney, resulting in decreased renal functioning and metabolism. Loss of podocyte insulin signaling results in glomerular injury (106); however, the role of fenestration loss remains to be studied.

### Sepsis

GEnC fenestration loss is seen in sepsis with bacterial lipopolysaccharide and TNF treatment significantly reducing fenestral density while increasing fenestral diameter (107). This is considered to play a key role in the pathogenesis of sepsis-induced acute kidney injury.

Fenestration loss in LSECs in response to toxins is a proposed defense mechanism to protect the entry of toxins into the space of Disse and prevent hepatocyte injury (108). GEnC fenestrae may play a similar role in the kidneys. As the kidneys serve as a natural blood filter, there is great potential for nephrotoxicity. Fenestration loss associated with toxins, such as bacterial endotoxin in sepsis (107), may offer a protective mechanism to prevent further downstream damage to the nephron rather than being a direct result of toxin injury. Regardless, loss of fenestrations will have implications for the delivery of solutes and electrolytes to renal tubules and also in fluid filtration and regulation.

### Thrombotic Microangiopathy

The pathologies of thrombotic microangiopathy and preeclampsia are similar and result in GEnC injury, fenestration loss, and abnormal renal filtration function. Abnormal glomerular VEGF-A signaling is central to the development of these pathologies. Renal thrombotic microangiopathy-type glomerular lesions have been identified in a mouse model in which there is dual knockout of EHD3 and EHD4 (57). EHD3 and EHD4 are involved in endocytic recycling, and it has been postulated that EHD3 and EHD4 may be involved in the endocytic trafficking of VEGFR2 in GEnCs (57). Indeed, decreased VEGFR2 expression in GEnCs was demonstrated in EHD3 and EHD4 knockout mice (57). VEGF dysregulation plays a key role in the etiology of preeclampsia (109). Placental soluble fms-like tyrosine kinase 1 (sFlt-1), a splice variant of VEGF, antagonizes VEGF binding and specifically VEGFA/VEGFR2 binding in the glomerulus (109). The endothelial injury and fenestration loss associated with increased sFlt-1 are more severe in mice deficient in endothelial nitric oxide (110). This was suggested to be related to an activated endothelin-1 system (110). Treatment with ambrisentan (an endothelin receptor type A antagonist) prevented fenestration loss (110, 111).

Thrombotic microangiopathy can also be identified in patients with hemolytic uremic syndrome secondary to Shiga toxin-producing *Escherichia coli* infection. Activation of complement cascade is considered a key mechanism in its development. Atypical hemolytic uremic syndrome associated with genetic defects has also been reported (112).

### Other Kidney Diseases

Transplant glomerulopathy is associated with kidney graft dysfunction and reduced graft survival. Loss of fenestrations is identified in these patients but is seen at a late stage of pathology following GEnC injury (113). In a mouse model of glomerulonephritis, persistence of GEnC cell injury including loss of fenestrations was found to be SMAD3 dependent (114). SMAD3 is a key signaling molecule for transforming growth factor (TGF)-β, with TGF-β being a known angiogenic growth factor important in endothelial health (111). Fabry’s disease, in which patients have an absence of α-galactosidase resulting in fat build up in renal cells, is associated with a decreased percentage of fenestrated endothelium (115). In addition, patients with Denys–Drash syndrome with loss of the glomerular antiangiogenic VEGF_165_b isoform also demonstrate fenestration loss (116). Fenestration loss in LSECs is associated with aging (15, 39, 117) and is a proposed mechanism for dyslipidemia in elderly people. It is therefore plausible that the age-related decline in GFR might also be related to loss of GEnC fenestrations, although this has never been studied. Whether aging per se is a disease is debatable; however, age-related loss of fenestrations may be important as a potential contributor to the decline in GFR.

## STUDYING GEnC FENESTRATIONS

**Figure 6.**
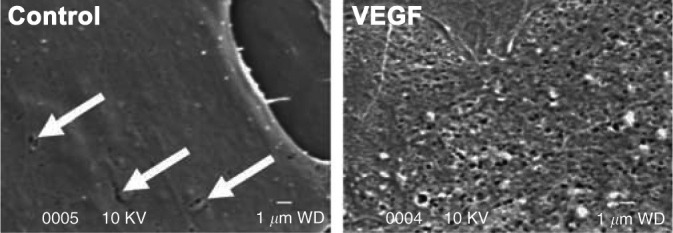
Scanning electron micrograph showing possible fenestrations in a conditionally immortalized glomerular endothelial cell line in untreated cells (white arrows) and cells treated with vascular endothelial growth factor (VEGF). Organized sieve plates are not apparent. Scale bar = 1 µm. (Taken from Ref. 118.)

**Figure 7.**
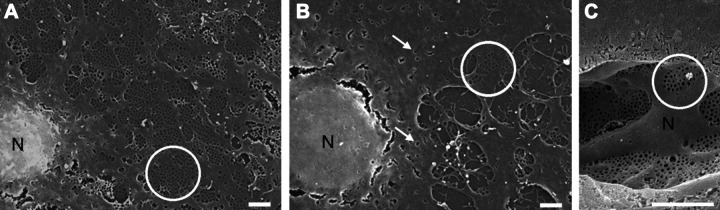
Scanning electron micrographs showing loss of fenestrations and artifacts that may appear as fenestrations in liver sinusoidal endothelial cells (LSECs). *A*: freshly isolated LSEC with well-defined fenestrations organized in sieve plates (white circle). *B*: LSEC after 1 day in culture. The cells are largely defenestrated. The arrow indicates transcytoplasmic holes considered to be artifacts, and the white circle indicates remaining sieve plates. *C*: liver sinusoids with fenestrae present in organized sieve plates. Scale bars = 2 µm. (Taken from Ref. 119.)

A significant barrier to overcome in further understanding of the cellular mechanisms that regulate GEnC fenestrations is the lack of an ideal in vitro model. A single-monolayer endothelial cell culture system does not accurately reflect the complex biological in vivo glomerular environment. A conditionally immortalized GEnC line (Fig. 6; 118) and human primary GEnCs (95) have been shown to possess possible fenestrations. However, the fenestrations appear rudimentary, and organized sieve plates, as seen in vivo, are not identifiable. To add further confusion, it has been shown in LSECs that the processing of samples in preparation for scanning electron microscopy (SEM) can result in holes within the cytoplasm that may have the appearance of fenestrations (Fig. 7; 119). Similar holes can also be seen in cells cultured over a long period of time. Fenestrae are considered to be rapidly lost when grown in culture, with organized sieve plates disappearing after ∼1 day in culture in LSECs (60, 119). More importantly, it is unclear how well cell culture models of GEnCs reflect the biological processes in vivo. TNF treatment of human GEnCs in culture resulted in smaller but more numerous fenestrations (120). These findings are in direct contradiction to those reported in vivo, in which TNF infusion resulted in decreased fenestrae number and increased width (107).

It is recognized that once endothelial cells are removed from their native vascular bed and grown in conventional single cell-type monoculture they will lose their extracellular regulators and undergo phenotypic drift (121). The endothelial heterogeneity that exits in vivo is lost as endothelial cells are repeatedly passaged (122). Peritubular endothelial cells have been shown to lack the molecular signature linked to growth capabilities compared with human umbilical vein endothelial cells, leading to the conclusion that different endothelial cells have the potential to better survive in culture than others (19). In addition, protocols for isolating endothelial cells often do not give 100% purity. All these factors are likely to influence achieving a fenestrated phenotype for GEnCs in monoculture.

Other endothelial cell lines that readily form fenestrations in culture, such as bEND5 cells (58, 65), have been studied to understand fenestration formation and regulation. However, how well the biology of this cell type translates to GEnCs given the vast heterogeneity between endothelial cells is unclear. Culture systems comprising different cell types that are present in the glomerulus, thus providing opportunity for cross talk mechanisms, offer potential. Indeed, a triculture system demonstrated improved maintenance of the endothelial phenotype in vitro for at least 3 wk when LSECs were cultured with primary human hepatocytes and fibroblasts (123). More recently, microfluidic “organ on a chip” systems have been reported as models for studying the glomerulus. These advanced platforms are considered to more closely mimic physiological systems, including by incorporating continuous medium flow than can be achieved in standard cell culture. They have the potential to provide the critical mechanical (e.g., shear stress) and angiogenic factors required for vascularization (124). A further advantage is that endothelial cells can be cocultured with other cells allowing for cell-to-cell signaling. There are several studies that have developed microfluidic “glomerulus on a chip” systems for studying the filtration barrier; however, none have reported the presence of GEnC fenestrations in these models (125–128). Human-induced pluripotent stem cells have been used to develop kidney organoids (129). However, in vitro cultured organoids do not show evidence of vasculature formation. Transplantation in vivo can promote the formation of glomerular and peritubular capillary beds, but these are not fenestrated (130). Progressive maturation of organoids following transplantation under the renal capsule with demonstratable vasculature perfusion is required for the formation of a fenestrated glomerular endothelium (131).

**Figure 8.**
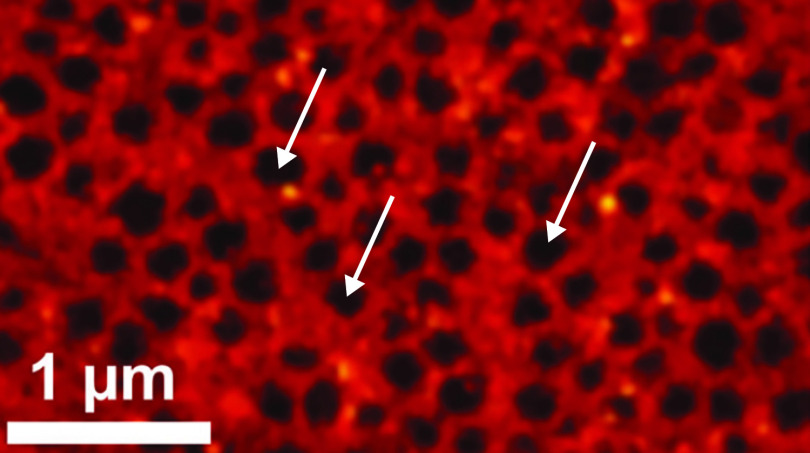
Direct stochastic optical reconstruction microscopy showing liver sinusoidal endothelial cell fenestrae organized in sieve plates. Red staining represents the cell membrane, and the negatively stained areas are empty fenestrations (white arrows; 132).

The small size of endothelial cell fenestrations is beyond the limit of resolution of light microscopy. Traditionally, their visualization has required the use of electron microscopy such as SEM and transmission electron microscopy (TEM) to provide the necessary high resolution. SEM allows visualization of large areas of the endothelial cell surface for observing sieve plates and measuring large numbers of fenestrations. SEM is the primary electron microscopy method for studying LSEC fenestrations, and there are published protocols for standardizing fenestration diameter, frequency, and porosity measurements (2). In the absence of obvious sieve plates, it can be difficult to confirm with SEM whether the pore that is visualized is a complete transcytoplasmic pore that distinguishes a fenestration or an invagination of the cell membrane as would be seen in a caveolae. TEM can differentiate such structures as the complete discontinuity of the cytoplasm can be visualized. TEM, however, does have the disadvantage that the number of fenestrations that can be analyzed in cross section is limited. Newer, more advanced imaging techniques known as super resolution microscopy may offer the potential to facilitate the study of fenestrations. SIM has been used to visualize LSEC fenestrations (63). An advantage of SIM is that it can be used with any conventional fluorophore and cell preparation is identical to other established light microscopy methods. This technique offers improved resolution of the fenestrations compared with light microscopy; however, the resolution is not as great as SEM. Many imaging techniques incorporate the use of fluorescently labeled proteins; however, specific markers for GEnC fenestrations have not yet been identified, making it difficult to validate such techniques to study these structures. Direct stochastic optical reconstruction microscopy has been used in visualizing LSEC fenestrations (132) and offers a potential solution in this respect. This technique incorporates a cell membrane label and the fenestrations are visualized as areas of negative contrast (without label; Fig. 8). As mature GEnCs are empty pores, the use of such a negative contrast technique would offer potential for their study and offer advantages over SEM or TEM in terms of time of processing and analysis and costs. However, its application is best suited to in vitro models that are currently lacking for GEnC fenestrae.

## CONCLUSIONS

There is increasing evidence regarding the role of the glomerular endothelium as a key player in the development and progression of kidney diseases. Loss of fenestrations is an important characteristic of GEnC damage and contributes to a decline in filtration function. Surprisingly, there is a paucity of knowledge related to the fundamental biology of the regulation of GEnCs. This is largely hampered by the lack of a reliable in vitro model in which to study them. In addition, knowledge of factors influencing GEnC fenestrations requires accurate measurement of the density and size of fenestrations requiring ultrastructural studies with high-resolution imaging techniques. Understanding fenestration regulation provides an important target for modulation leading to the development of drugs aimed at preventing GEnC fenestration loss. Such therapeutics offer the potential to prevent declining GFR and subsequent progressive renal injury related to fenestration loss.

## GRANTS

N.C.F. was funded by Wellcome Trust Grant 104507/Z/14/Z. 

## DISCLOSURES

No conflicts of interest, financial or otherwise, are declared by the authors. 

## AUTHOR CONTRIBUTIONS

N.C.F. prepared figures; N.C.F. drafted manuscript; N.C.F., C.R.N., G.I.W., R.R.F., and S.C.S. edited and revised manuscript; N.C.F., C.R.N., G.I.W., R.R.F., and S.C.S. approved final version of manuscript. 
